# Effects of Temperature and Photoperiod on the Immature Development in *Cassida rubiginosa* Müll. and *C*. *stigmatica* Sffr. (Coleoptera: Chrysomelidae)

**DOI:** 10.1038/s41598-019-46421-3

**Published:** 2019-07-11

**Authors:** Dmitry Kutcherov, Elena B. Lopatina, Stepan Yermakov

**Affiliations:** Department of Entomology, St. Petersburg State University, 7–9 Universitetskaya nab., St. Petersburg, 199034 Russia

**Keywords:** Agroecology, Ecophysiology, Entomology

## Abstract

Tortoise beetles (*Cassida* and related genera) are a large cosmopolitan group that includes several pests of agricultural crops and natural enemies of weeds but their biology and ecology remain poorly known. Using a set of environmental chambers, we address simultaneous effects of temperature and photoperiod on immature development and adult body mass in two European species, *C*. *rubiginosa* and *C*. *stigmatica*. Consistent with its broader distribution range, the former species is less susceptible to low rearing temperatures, develops faster and has a larger body mass than the latter. However, *C*. *rubiginosa* seems to be less adapted to late-season conditions as a short-day photoperiod accelerates its immature development to a lesser extent than it does in *C*. *stigmatica*, which nevertheless results in greater larval mortality and slightly but significantly smaller adults. By contrast, in *C*. *stigmatica*, which is more likely to encounter late-season conditions due to its slower life cycle, short-day acceleration of development is achieved at no cost to survivorship and final body mass. The experiment with *C*. *stigmatica* was repeated during two consecutive years with different methods and the main results proved to be well reproducible. In addition, laboratory results for *C*. *rubiginosa* agree with field data from literature.

## Introduction

Tortoise beetles are a charismatic and diverse clade within the subfamily Cassidinae (Coleoptera: Chrysomelidae) and comprise about 3000 described species^1,2^. Adults are easily recognizable by their explanate pronotum and elytra, and larvae of most species have a pair of caudal processes which carry a protective shield constructed of feces and/or shed exuviae. As with many (if not all) insect groups, there is an immense body of systematic and faunal studies on tortoise beetles, but little is known about their life histories and ecology in general, especially outside the Holarctic region. While some ecological aspects have received modest attention, e.g., host plant associations^3,4^ and defenses against predators^5–7^, physiological reactions of tortoise beetles to basic abiotic factors remain decidedly understudied. This is especially lamentable in view of potential importance of some species as natural enemies of invasive weeds^8,9^.

## Developmental Responses To Temperature In Tortoise Beetles

To the best of our knowledge, the effects of temperature manipulations on development have been published for six species of tortoise beetle: *Cassida nebulosa*^10^, *C*. *rubiginosa*^11^, *Chelymorpha cribraria*^12^, *Gratiana boliviana*^13^, *G*. *graminea*^14^ and *Metriona elatior*^15^. Unfortunately, reported developmental rates for *C*. *nebulosa* and *G*. *graminea* strongly depart from a linear relationship with temperature, which is not expected to occur in the non-stressful thermal range^16^. Data for *C*. *cribraria* and *M*. *elatior* are incomplete as only two rearing temperature values are available per species and these are not enough to plot a regression line. Thus, sufficiently detailed and comparable data on temperature-dependent development exist for only two tortoise beetle species, *C*. *rubiginosa* and *G*. *boliviana*.

Absolute differences in developmental rates of *C*. *rubiginosa* and *G*. *boliviana* are fairly small: the period from oviposition to adult eclosion lasts, depending on rearing temperature, from less than 3 weeks to over 1.5 months in both species^11,13^. However, the lower temperature threshold for total immature development is shifted leftward in the northern species *C*. *rubiginosa* (10.4 °C)^11^ relative to that in tropical *G*. *boliviana* (13.7 °C)^13^. Slopes of the developmental rate-temperature relationship are also different with *C*. *rubiginosa* being less temperature-sensitive (the slope is shallower) than its counterpart from a warmer climate. In other words, the north temperate species develops more rapidly than the tropical one at low temperatures, both species are on a par in the midrange, but at high temperatures the tropical species is faster. Such mutual position of thermal reaction norms for development is quite often observed along latitudinal gradients at the interspecific level in various ectothermic animals and in plants^17–20^ and, arguably, mirrors adaptation of the life histories to climatic conditions. This example illustrates how informative it can be to examine development at several controlled temperatures and what ecological insights can be gained from such experimental designs.

In addition to the existence of interspecific differences, individuals of the same species may also differ in their developmental or growth rate and its relation to temperature, e.g., as a response to a change in the environment. Diet, photoperiod, humidity, diapause commitment, and other extrinsic and intrinsic factors are known to modify thermal reaction norms for development and growth in a number of insect species^21–26^. No such studies have ever been conducted with either tortoise beetles or other Cassidinae, although it is conceivable that this predominantly tropical group likely faced numerous adaptive challenges during colonization of higher latitudes and could have evolved various kinds of developmental plasticity in seasonal climates. To begin filling the mentioned gaps in cassidine ecophysiology, we have experimentally studied simultaneous effects of temperature and photoperiod on immature development in two commonest European species belonging to the genus *Cassida*.

## Study Species

*Cassida rubiginosa* O. F. Müller is a medium-sized beetle, 6.0–8.0 mm in length, indigenous to a vast territory in the Palaearctic region from Great Britain and the Mediterranean through Siberia, Kazakhstan and Mongolia to the Kuril Islands and Japan^27–32^. It was accidentally introduced in North America, where it rapidly naturalized and spread – sometimes with human aid, because it was viewed as a potential biocontrol agent against invasive thistles^11^. Recently, *C*. *rubiginosa* was also released in New Zealand for the same purpose^33^. In both new locations, the beetle alone only slightly reduced thistle performance, although its effect may be stronger in conjunction with other natural enemies and competitors^34,35^. Throughout its distribution range, *C*. *rubiginosa* usually prefers open, moderately humid habitats including fields and pastures, abandoned farmland, meadows, banks and disturbed areas, but can also be found in forests^31,34,36–38^. Besides *Carduus* and *Cirsium* thistles, larvae and adults feed on a wide variety of other asteraceous plants from the tribe Cynareae (=Cardueae), such as species of *Arctium*, *Cynara*, *Silybum etc*.^8,27,28,37–39^.

*Cassida stigmatica* Suffrian has a smaller body 5.5–6.3 mm in length^30^, more restricted geographic occurrence, and narrower host plant spectrum than *C*. *rubiginosa*. Its distribution range extends from Europe to southern Siberia and Central Asia, reaching eastward to Lake Baikal and the Chinese Tian Shan^28,32,40^. The species inhabits dry meadows, sparse forests, sandy banks, wastelands, roadsides *etc*., where adults and larvae feed on *Tanacetum* spp. and seldom on *Achillea* spp^28,32,38,40,41^. As an intriguing exception, this species is reported to develop on Chenopodiaceae and feed as adults on *Artemisia* in a dry steppe in Kazakhstan^42^. In Europe, *C*. *stigmatica* seems to be spreading and becoming more common. Reitter^43^ in his famous Fauna Germanica refers to this beetle as an infrequent montane species. A century later, *C*. *stigmatica* can be collected in Germany’s northern lowland, and this is indeed where most experimental ecological research on it has been done so far, including evaluation of this herbivore for its efficacy against invasive tansy *T*. *vulgare*^9,44^.

The life cycles of *C*. *rubiginosa* and *C*. *stigmatica* are typical of Holarctic tortoise beetles^37,38,41^. All developmental stages occur openly on the host plant. Adults emerge in the spring, feed on young foliage and mate. Eggs are usually deposited on the underside of the leaves, singly or in small groups, and are protected with an oothecal covering. Larvae pass through five instars, and the cuticle shed during each molt remains attached to the caudal processes. Larvae of *C*. *rubiginosa*, in addition, defecate onto these processes so that a massive exuvio-fecal shield is formed, which is usually discarded before pupation, whereas *C*. *stigmatica* larvae carry only a train of exuviae, which is retained in the pupa^45^. Adults of the new generation emerge during summer and, after feeding, move to overwintering quarters. They spend winter in leaf litter, under brushwood and trees *etc*., preferably in the elevated, drier parts of the landscape^38^. Both species are univoltine regardless of climate^11,33,37,38,41^. In the greenhouse, *C*. *rubiginosa* can break quiescence and produce a second generation the same year^11^, but this seems to be never achieved under natural conditions. Adults of *C*. *rubiginosa* are long-lived and can overwinter as many as three times^31,37^.

## Hypothesis

Adult tortoise beetles have a protracted oviposition period, and preimaginal development takes several weeks in the field^37^. Therefore, external conditions will differ among clutches and fluctuate during individual life, and it would be advantageous if the offspring was able to cope with this environmental variation. As short-day photoperiods and low temperatures signal the approaching of autumn, we expected that developmental rate and body size in *C*. *rubiginosa* and *C*. *stigmatica* would respond to these conditions so as to ensure a timely completion of development and successful overwintering.

## Results

### Survivorship and sex ratio

**Figure 1:**
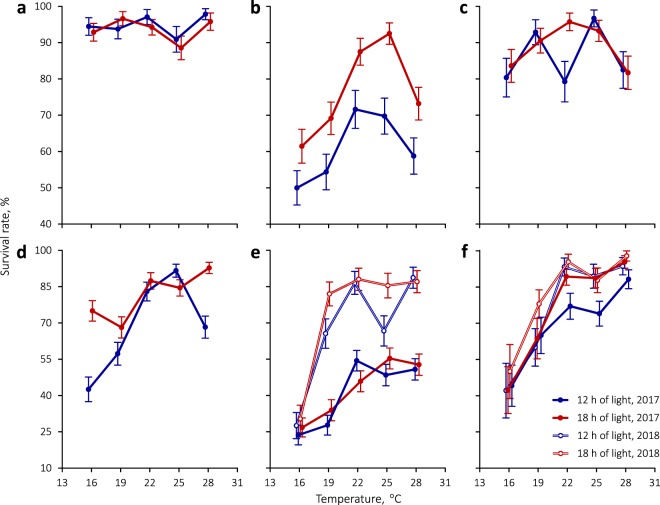
Survivorship of (**a**) eggs, (**b**) larvae, and (**c**) pupae of *C*. *rubiginosa* and (**d**) eggs, (**e**) larvae, and (**f**) pupae of *C*. *stigmatica* under different combinations of temperature and photoperiod. Symbols are slightly set apart along the temperature axis for clarity. Vertical bars denote binomial s.e.

Eggs and pupae of *C*. *rubiginosa* had high survival rates, which were independent of temperature and photoperiod (Fig. 1a,c; Table 1). By contrast, larval survivorship in *C*. *rubiginosa* was significantly affected by both factors (Fig. 1b, Table 1). Larvae fared best at 22–25 °C under both photoperiods. Short-day conditions had a negative impact on larval survival at all the temperatures tested (Fig. 1).

**Table 1:** Summary of generalized linear models (GLMs), using binomial errors and a logit link function, of predictors for immature survival and adult sex ratio in two *Cassida* species.

	Egg hatch			Larval survival			Pupal survival			Adult sex ratio		
	df res.	LRT χ^2^	*P*	df res.	LRT χ^2^	*P*	df res.	LRT χ^2^	*P*	df res.	LRT χ^2^	*P*
***C***. ***rubiginosa***												
Temperature	882	0.03	0.9	943	15.1	0.0001	636	0.1	0.7	559	0.4	0.5
Photoperiod	882	0.9	0.3	943	26.6	<0.00001	636	0.9	0.3	559	0.02	0.9
***C***. ***stigmatica***												
Temperature	1059	55.6	<0.00001	1832	129.3	<0.00001	926	106.2	<0.00001	741	1.7	0.2
Photoperiod	1059	24.4	<0.00001	1832	0.3	0.6	926	9.4	0.002	741	0.5	0.5
Experimental year	—	—	—	1832	130.3	<0.00001	926	6.6	0.01	741	1.0	0.3

LRT, likelihood ratio test. Model (numerator) degrees of freedom equal 1 for all terms. Residual (denominator) degrees of freedom are greater for larvae than those for eggs because experimental larvae originated from two sources (for details, see the *Experimental design* section). Egg development experiment was not repeated during the second year.

Temperature and photoperiod significantly influenced *C*. *stigmatica* survivorship (Table 1). There was a tendency for survival rates to be lower in cooler treatments and under short-day conditions (Fig. 1d–f), although there were quite a few exceptions to the latter pattern. For example, in the 2017 experiment, larvae actually survived better under short-day conditions (Fig. 1e), which is reflected in a significant photoperiod by year interaction in GLMs (likelihood ratio test χ^2^ = 19.2, *P* = 0.00001). Maintenance of *C*. *stigmatica* larvae on living plants instead of cut leaves during the second experimental year significantly improved larval and, to a lesser extent, pupal survival (Fig. 1e,f, Table 1). In larvae, this ameliorating effect was observed at all but the lowest temperature of 16 °C, hence a significant temperature by year interaction (χ^2^ = 5.0, *P* = 0.03).

Interaction terms in GLMs were generally non-significant with *P*-values of 0.5 and higher, but, when these interaction terms were included in the models, main effects tended to become non-significant as well. The model for *C*. *stigmatica* larvae mentioned above was the only exception where inclusion of double interactions did not affect the output; however, even in that case, addition of a triple temperature × photoperiod × year interaction (χ^2^ = 0.3, *P* = 0.6) rendered all of the remaining effects non-significant (χ^2^ < 0.5, *P* > 0.5). Therefore, only the summaries of main-effects models are provided in Table 1.

The adult sex ratio in both species varied among the experimental regimens (Supplementary Tables S1 and S2) but this variation was not associated with rearing conditions (Table 1), suggesting that mortality during development was unrelated to sex.

### Egg development

**Figure 2:**
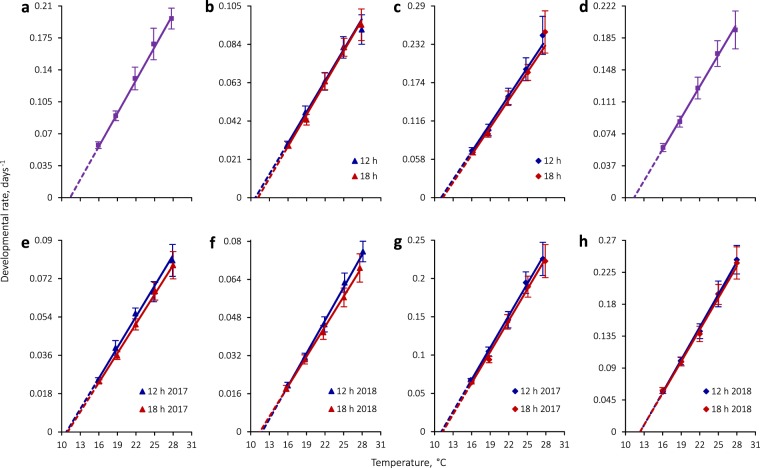
Thermal reaction norms for (**a**) egg, (**b**) larval, and (**c**) pupal development in *C*. *rubiginosa* and (**d**) egg, (**e**,**f**) larval, and (**g**,**h**) pupal development in *C*. *stigmatica*. Regression lines are plotted based on GLS model parameters fit by REML. Symbols with bars denote mean developmental rates ± s.d., which were not used in fitting the regression models and are shown for illustration purposes; position of the symbols on the temperature axis matches actual temperatures during the experiments. Egg development data were pooled between two photoperiodic regimens.

Egg developmental rates linearly increased with temperature and were very similar in *C*. *rubiginosa* and *C*. *stigmatica* (Fig. 2a,d; Tables 2–6; Supplementary Fig. S1). There was a marginally significant effect of photoperiod, according to GLS models (Table 4), but the absolute differences between photoperiodic regimens were negligibly small, except at 16 °C, and inconsistent (Tables 2 and 3). Therefore, egg development data were pooled between photoperiodic regimens. There was no significant difference in egg developmental rates between *C*. *rubiginosa* and *C*. *stigmatica*, according to a GLS ANOVA with species and temperature as the only two predictor variables (effect of species: *F*_1,1629_ = 0.2, *P* = 0.7). Lower temperature thresholds for egg development in these two species coincided and the sum of degree-days was almost identical (Tables 5 and 6).

**Table 2:** Duration of immature stages (mean ± s.d., days) of *C*. *rubiginosa* under five constant temperatures and two photoperiods.

Temperature, °С		Day length, h	Eggs	*N*	Larvae	Pupae	*N* ^†^
Set	Real						
16	15.9	12	18.3 ± 0.81	85	34.6 ± 2.58	14.2 ± 1.09	45
	16.1	18	16.8 ± 0.89	104	35.0 ± 2.11	14.6 ± 0.82	56
19	18.7	12	10.9 ± 0.66	75	21.6 ± 2.21	9.8 ± 0.74	52
	18.9	18	11.5 ± 0.54	85	23.5 ± 2.05	10.4 ± 0.54	67
22	21.9	12	7.9 ± 0.76	66	15.8 ± 1.32	6.6 ± 0.56	42
	21.9	18	7.6 ± 0.74	114	15.8 ± 1.31	6.7 ± 0.49	67
25	24.9	12	6.1 ± 0.75	60	12.2 ± 0.92	5.2 ± 0.43	58
	25.0	18	5.9 ± 0.59	85	12.2 ± 0.73	5.3 ± 0.35	69
28	27.7	12	5.1 ± 0.34	90	10.9 ± 0.97	4.1 ± 0.44	47
	27.9	18	5.1 ± 0.24	68	10.7 ± 1.08	4.0 ± 0.47	58

^†^Larval and pupal sample sizes are equal because only individuals that survived to the adult stage were taken into account.

**Table 3:** Duration of immature stages (mean ± s.d., days) of *C*. *stigmatica* under five constant temperatures and two photoperiods.

Year and rearing method	Temperature, °С		Day length (h)	Eggs	N	Larvae	Pupae	N^†^
	Set	Real						
2017 (larvae kept in cups, fed with cut leaves)	16	15.9	12	18.3 ± 1.16	40	40.9 ± 1.76	14.9 ± 0.46	15
		16.1	18	16.8 ± 1.20	78	42.2 ± 1.36	15.5 ± 0.48	11
	19	18.7	12	11.2 ± 0.86	63	25.5 ± 2.78	9.6 ± 0.51	26
		18.9	18	11.6 ± 0.65	77	28.0 ± 1.38	10.6 ± 0.47	21
	22	21.9	12	8.6 ± 0.74	78	18.0 ± 0.84	6.9 ± 0.50	47
		21.9	18	7.4 ± 0.38	83	19.9 ± 1.05	7.0 ± 0.45	67
	25	24.9	12	6.1 ± 0.54	98	15.3 ± 1.11	5.2 ± 0.36	54
		25.1	18	6.0 ± 0.57	98	15.2 ± 0.96	5.3 ± 0.41	55
	28	27.7	12	5.1 ± 0.33	71	12.5 ± 1.27	4.5 ± 0.46	60
		28.0	18	5.3 ± 0.67	115	12.9 ± 1.16	4.5 ± 0.47	62
2018 (larvae kept in plastic glasses with living host plants)	16	16.0	12	—	—	51.2 ± 2.90	17.7 ± 0.80	8
		15.8	18	—	—	55.1 ± 3.88	17.2 ± 1.20	10
	19	18.8	12	—	—	31.9 ± 1.67	10.0 ± 0.53	24
		18.8	18	—	—	33.1 ± 2.17	10.1 ± 0.58	39
	22	21.9	12	—	—	22.3 ± 1.64	7.1 ± 0.53	42
		21.8	18	—	—	24.1 ± 1.80	7.3 ± 0.56	42
	25	25.1	12	—	—	16.0 ± 1.07	5.2 ± 0.53	34
		25.0	18	—	—	17.8 ± 1.28	5.2 ± 0.42	36
	28	28.1	12	—	—	13.2 ± 0.79	4.1 ± 0.37	44
		27.7	18	—	—	14.6 ± 1.34	4.2 ± 0.45	46

Egg development experiment was not repeated during the second year. ^†^Larval and pupal sample sizes are equal because only individuals that survived to the adult stage were taken into account.

**Table 4:** Results of generalized least squares ANOVA of the effects of temperature, photoperiod, sex, experimental year, and their interactions on developmental rate and body mass in two *Cassida* species.

	Egg developmental rate			df res.	Larval developmental rate		Pupal developmental rate		Adult body mass	
	df res.	F	*P*		F	*P*	F	*P*	F	*P*
***C***. ***rubiginosa***										
Temperature	828	20873.7	<0.00001*	553	19849.5	<0.00001*	14326.2	<0.00001*	8.9	0.003
Photoperiod	828	3.4	0.07	553	35.3	<0.00001*	70.6	<0.00001*	17.5	0.00003*
Sex	—	—	—	553	11.7	0.0007*	1.5	0.2	624.5	<0.00001*
Temperature × photoperiod	828	0.3	0.6	553	3.1	0.08	0.7	0.4	1.1	0.3
Temperature × sex	—	—	—	553	0.0003	1.0	0.1	0.7	0.9	0.3
Photoperiod × sex	—	—	—	553	0.3	0.6	9.0	0.003	6.9	0.009
Temperature × photoperiod × sex	—	—	—	553	0.01	0.9	3.6	0.06	1.2	0.3
***C***. ***stigmatica***										
Temperature	797	18966.6	<0.00001*	727	32628.0	<0.00001*	42063.5	<0.00001*	10.1	0.002
Photoperiod	797	8.3	0.004	727	82.7	<0.00001*	112.1	<0.00001*	3.1	0.08
Sex	—	—	—	727	3.4	0.07	0.4	0.6	858.6	<0.00001*
Experimental year	—	—	—	727	1130.3	<0.00001*	406.2	<0.00001*	296.1	<0.00001*
Temperature × photoperiod	797	0.2	0.7	727	28.9	<0.00001*	8.0	0.005	0.03	0.9
Temperature × sex	—	—	—	727	6.6	0.01	0.07	0.8	0.2	0.7
Temperature × experimental year	—	—	—	727	33.1	<0.00001*	93.3	<0.00001*	3.1	0.08
Photoperiod × sex	—	—	—	727	1.8	0.2	0.7	0.4	2.6	0.1
Photoperiod × experimental year	—	—	—	727	10.5	0.001	4.5	0.03	0.0	1.0
Sex × experimental year	—	—	—	727	2.7	0.1	0.8	0.4	7.1	0.008
Temperature × photoperiod × sex	—	—	—	727	1.4	0.2	1.7	0.2	0.8	0.4
Temperature × photoperiod × experimental year	—	—	—	727	17.1	0.00004*	0.02	0.9	0.05	0.8
Temperature × sex × experimental year	—	—	—	727	0.2	0.6	9.2	0.003	0.1	0.8
Photoperiod × sex × experimental year	—	—	—	727	6.1	0.01	0.2	0.7	0.02	0.9
Temperature × photoperiod × sex × experimental year	—	—	—	727	10.8	0.001	7.3	0.007	2.6	0.1

Model (numerator) degrees of freedom equal 1 for all terms; residual (denominator) degrees of freedom are equal for larvae, pupae, and adults because only individuals that survived to the adult stage were taken into account. Sex could not be inferred for eggs because hatchlings were kept in groups and it was not subsequently possible to match individual adults with individual eggs. Egg development experiment was not repeated during the second year. *Due to large sample sizes, only effects with *p* < 0.001 were considered significant.

**Table 5:** Generalized least squares regression parameters (±s.e.) for temperature-dependent development in *Cassida rubiginosa*, fit by restricted maximum likelihood.

Stage	Day length, h	*a*, d^−1^	*b*, °C^−1^ × d^−1^	LTT, °C	SDD, °C × d
***C***. ***rubiginosa***					
Eggs	—	−0.1372 ± 0.00113	0.0121 ± 0.00006	11.4	82.7
Larvae	12	−0.0609 ± 0.00122	0.0057 ± 0.00007	10.7	175.9
	18	−0.0646 ± 0.00097	0.0058 ± 0.00005	11.1	172.5
Pupae	12	−0.1558 ± 0.00351	0.0141 ± 0.00020	11.0	70.9
	18	−0.1577 ± 0.00266	0.0139 ± 0.00015	11.3	71.9

LTT, lower temperature threshold for development. SDD, sum of degree-days.

**Table 6:** Generalized least squares regression parameters (±s.e.) for temperature-dependent development in *Cassida stigmatica*, fit by restricted maximum likelihood.

Stage	Day length, h	Year and larval rearing method	*a*, d^−1^	*b*, °C^−1^ × d^−1^	LTT, °C	SDD, °C × d
***C***. ***stigmatica***						
Eggs	—	—	−0.1383 ± 0.00166	0.0121 ± 0.00009	11.4	82.4
Larvae	12	2017 (cups, cut leaves)	−0.0517 ± 0.00101	0.0048 ± 0.00005	10.8	207.8
	12	2018 (glasses, living plants)	−0.0547 ± 0.00099	0.0046 ± 0.00005	11.9	216.9
	18	2017 (cups, cut leaves)	−0.0503 ± 0.00087	0.0046 ± 0.00004	11.0	217.6
	18	2018 (glasses, living plants)	−0.0483 ± 0.00115	0.0042 ± 0.00006	11.5	238.1
Pupae	12	2017 (cups, cut leaves)	−0.1544 ± 0.00199	0.0139 ± 0.00012	11.1	71.9
	12	2018 (glasses, living plants)	−0.1897 ± 0.00310	0.0153 ± 0.00016	12.4	65.2
	18	2017 (cups, cut leaves)	−0.1544 ± 0.00220	0.0136 ± 0.00013	11.3	73.4
	18	2018 (glasses, living plants)	−0.1839 ± 0.00463	0.0149 ± 0.00023	12.3	67.1

LTT, lower temperature threshold for development. SDD, sum of degree-days.

### Larval development

Larval developmental rates showed a linear relationship with temperature in the studied range (Fig. 2b,e,f; Tables 5 and 6; Supplementary Fig. S1). Apart from the strongly pronounced effect of temperature, larval development in *C*. *rubiginosa* was significantly influenced by photoperiod and sex (Table 4). In particular, larvae developed slightly faster under short-day conditions at the two lower temperatures than in the corresponding long-day regimens (Table 2). However, despite the seemingly different response to photoperiod at different temperatures, there was no significant temperature by photoperiod interaction, which meant that the slope of the developmental rate-temperature relationship was unaffected by photoperiod (Fig. 2b, Table 5).

In *C*. *stigmatica*, larval development was significantly influenced by temperature, photoperiod, experimental year, and some interactions of these predictors (Table 4). Larval development was faster and the slope of the developmental rate-temperature relationship was steeper under short-day conditions relative to long-day conditions (Fig. 2e,f; Table 6), hence a significant photoperiod by temperature interaction (Table 4). Rearing on living plants during the 2018 experiment resulted in significantly slower development, shallower slopes of thermal reaction norms, and enhanced effect of photoperiod relative to the previous year’s results (Fig. 2e,f; Table 6), which was reflected in a significant triple interaction (Table 4).

In both species, larval males tended to develop more rapidly than females (Supplementary Tables S1 and S2), although only in *C*. *rubiginosa* this effect appeared significant (Table 4). There were no significant interaction terms including sex as a factor in the GLS models (*P* ≥ 0.001, Table 4), which meant that both sexes responded to temperature, photoperiod, and rearing method in a similar fashion, and so the developmental data in Tables 2, 3, 5, and 6 were pooled between sexes for the sake of brevity.

Lower temperature thresholds for larval development were similar in the two species and varied from 10.7 to 11.9 °C (Tables 5 and 6), but *C*. *rubiginosa* required a notably smaller sum of degree-days than *C*. *stigmatica*. In other words, larval development in the former species was more temperature-sensitive (the slopes of the thermal reaction norms were steeper) and uniformly faster than in the latter. This conclusion was well supported by a GLS ANOVA with species and temperature as predictor variables (2017 experiment, effect of species: *F*_1,975_ = 1613.3, *P* < 0.00001; temperature × species: *F*_1,975_ = 429.6, *P* < 0.00001).

### Pupal development

As with eggs, pupal developmental rates strongly depended on temperature and were similar in *C*. *rubiginosa* and *C*. *stigmatica*, but the former species tended to develop slightly faster (Fig. 2c,g,h; Table 2–6; Supplementary Fig. S1). Taking only the 2017 experiment into account, where *C*. *rubiginosa* and *C*. *stigmatica* could be compared directly because they were reared in identical conditions, there was a marginally significant difference in pupal developmental rates between the two species (GLS ANOVA, photoperiods combined, effect of species: *F*_1,975_ = 4.7, *P* = 0.03) but a highly significant temperature by species interaction (*F*_1,975_ = 20.1, *P* < 0.00001). Short-day conditions slightly but significantly accelerated pupal development in both species (Fig. 2c,g,h), although there did exist sporadic deviations from this general tendency in some experimental regimens (Tables 2 and 3). In addition, there was a significant temperature by experimental year interaction in the GLS model fit for *C*. *stigmatica* (Table 4), such that pupae in the 2018 experiment developed slower at low temperatures but faster at high temperatures than pupae in the previous year. In fact, lower temperature thresholds for pupal development differed more between experimental years in *C*. *stigmatica* than they did between photoperiods or even species (Tables 5 and 6).

### Adult body mass

**Figure 3:**
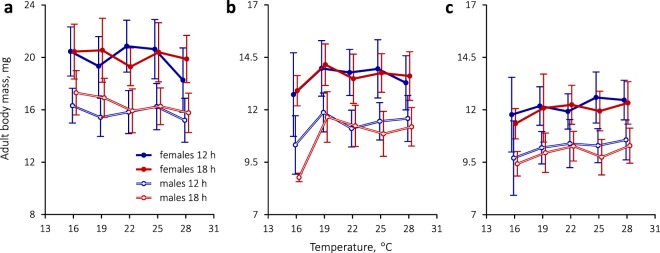
Thermal reaction norms for adult body mass in (**a**) *C*. *rubiginosa* and (**b**,**c**) *C*. *stigmatica* after rearing at different combinations of temperature and photoperiod. The latter species was tested during two consecutive years using different larval rearing methods, (**b**) in 2017, when larvae were confined to small cups and fed with cut leaves, and (**c**) in 2018, when larvae were maintained in plastic glasses and fed on living plants. Symbols with bars denote means ± s.d. and are slightly set apart along the temperature axis for clarity.

Regardless of experimental conditions, females of *C*. *rubiginosa* and *C*. *stigmatica* were significantly heavier than males (Table 4, Fig. 3). The effect of developmental temperature on adult body mass was subtle and only marginally significant. In *C*. *rubiginosa*, body mass was significantly affected by photoperiod, which was manifested in a weak tendency for relatively heavier adults under long-day conditions (Table 4, Fig. 3a). Across all temperature groups, *C*. *rubiginosa* males averaged at 15.8 mg under a short-day photoperiod and 16.4 mg under a long-day photoperiod; respective body mass values in females were 19.9 and 20.1 mg. In *C*. *stigmatica*, the effect of photoperiod on adult body mass was non-significant but there was a pronounced and significant difference between two experimental years (Table 4). Beetles reared on cut leaves in 2017 generally weighed more than those that emerged after developing on living plants in 2018 (Fig. 3b,c). On average across all experimental regimens in 2017, male *C*. *stigmatica* body mass was 11.0 mg and female body mass, 13.6 mg, whereas in 2018, mean male and female body mass only amounted to 10.1 and 12.1 mg, respectively. Even during the first experimental year, *C*. *stigmatica* was significantly smaller than *C*. *rubiginosa* (GLS ANOVA, effect of species: *F*_1,975_ = 1710.0, *P* < 0.00001). The largest of *C*. *stigmatica* females weighed about 16 mg and were approximately the size of average *C*. *rubiginosa* males.

## Discussion

Experimental rearing under different thermal and photoperiodic conditions reveals that immature survival, development and growth in tortoise beetles *C*. *rubiginosa* and *C*. *stigmatica* are sensitive to temperature and day length. However, the degree of this sensitivity varies considerably across species, developmental stage, and trait studied. Some of the responses were expected. For example, a sharp linear increase in developmental rate with rising temperature is a well-established phenomenon^16,46^. Acceleration of development by short-day conditions was also anticipated to occur as it is a widespread form of adaptive plasticity in response to seasonal time constraints. On the other hand, there are quite a few surprising findings which are more challenging to interpret. In particular, body mass in *C*. *stigmatica* and *C*. *rubiginosa* is largely independent of rearing temperature, contrary to a commonly observed negative size-temperature relationship. Small body size is often associated with rapid development but may alternatively be a consequence of disease, poor nutrition or stressful conditions, when development is prolonged. This is not the case here either, as *C*. *stigmatica*, which is significantly smaller than *C*. *rubiginosa*, develops significantly slower. Furthermore, *C*. *stigmatica* has even slower development and smaller body mass when reared under more favourable conditions.

### Temperature-dependent development

Egg development in *C*. *rubiginosa* and *C*. *stigmatica* proceeds at such a similar pace that it is not possible to separate these two species by egg development time at any of the temperatures tested. By contrast, pupal and especially larval development is faster in *C*. *rubiginosa* than *C*. *stigmatica*. The former species also has steeper thermal reaction norms in most cases (Fig. 2, Tables 5 and 6; Supplementary Fig. S1), which implies accumulation of fewer degree-days. All this is intriguing because *C*. *rubiginosa* is larger than *C*. *stigmatica* during all developmental stages. Usually, it is bigger insects that develop more slowly^47–51^ and accumulate a greater sum of degree-days^52^. Nevertheless, both life-history theory^49^ and evolutionary experiments^53^ show that selection can result in a faster body mass gain without increasing development time. Males of both species develop faster than females, albeit in *C*. *stigmatica* the difference is not statistically significant with our sample sizes. In any case, relatively faster male development is ubiquitous among the insects^54^.

In comparison with other leaf beetles (Chrysomelidae), whose thermal requirements for development are known, *C*. *rubiginosa* and *C*. *stigmatica* have slightly right-shifted lower developmental thresholds and smaller-than-average sums of degree days. In particular, an average studied leaf beetle requires 91.7 degree-days above 10.6 °C for egg development, 267.1 degree-days above 10.2 °C for larval and 96.3 degree-days above 10.1 °C for pupal development^16^. More experiments with different tortoise beetle species are needed to find out whether relatively high threshold and slope values (the sum of degree-days equals 1/*b*) are typical of this whole group.

### Temperature, sex, diet and body mass

Of all the factors studied, body mass in *C*. *rubiginosa* and *C*. *stigmatica* is mostly influenced by species identity and sex (Fig. 3, Table 4). While the effect of temperature is marginally significant and it is possible to discern weak tendencies in Fig. 3, differences between thermal regimens are too small to discuss them meaningfully. This finding conflicts with the widespread but poorly understood “temperature-size rule”, whereby most ectotherms attain larger size in cooler conditions^55^. Female beetles of both species are bigger than males, as is usual with insects^56^.

A smaller body mass in *C*. *stigmatica* is in line with hypotheses that animal species with narrower host plant ranges should comprise smaller individuals. However, opinions vary as to why this should be so and what comes first in such evolution: body size or diet breadth^47,57,58^. Also, while the latter two cited works do indeed show that larger moths have broader host plant repertoires, a study on butterflies^48^ finds no such relationship. We are inclined to agree with Wasserman and Mitter^57^ that a larger body size might enhance the generalists’ ability to cope with environmental variation and physiological stress. Not only is *C*. *rubiginosa* larger and less host-specific than *C*. *stigmatica*, but it also has a wider distribution range and, as our experiments show, better survives at low temperatures (Fig. 1). However, as *C*. *stigmatica* feeds on members of the tribe Anthemideae that contain high concentrations of monoterpenes which are responsible for their strong aromatic odors and insecticidal properties^59^, its smaller body size may as well be due to a greater investment of energy into detoxification of host plant defensive chemicals. Interestingly, larval development on living host plants during the 2018 experiment resulted in even smaller adult body mass, especially in females (Fig. 3b,c), relative to the previous year’s results when *C*. *stigmatica* was reared on cut leaves at a high relative humidity. The aim of the 2018 experiment was to improve rearing conditions and not to explicitly test for an inhibitory effect of host plant on herbivore development. It is not possible to quantify the confounding effects of genetic background and relative humidity which also differed between years. Nevertheless, it is worth noting that, while we did achieve higher survival rates, we obtained smaller adults with less pronounced sexual size dimorphism, which is actually indicative of poorer conditions^56^.

### Photoperiodic plasticity of developmental rate and body mass

Short-day photoperiod accelerates larval and pupal development in *C*. *rubiginosa* and *C*. *stigmatica*, albeit not strongly and not at all temperatures (Fig. 2, Table 2–4). In nature, both species complete only one generation per year and hibernate in the adult stage^37,38,41^. Therefore, relative acceleration of development as the season is waning and day length is decreasing apparently ensures that the overwintering stage is reached before the onset of cold weather and deterioration of host plants. The effect is especially pronounced in *C*. *stigmatica* larvae, which have the longest development time and thus face a higher risk of maturation at a suboptimal time of the year. Besides, short-day larvae of *C*. *stigmatica* have significantly steeper thermal reaction norms (Fig. 2e,f), i.e., their developmental rate is more sensitive to temperature change, which may be important in taking advantage of spells of warm weather late in the season. In *C*. *rubiginosa*, development under short-day conditions is accomplished at a cost of increased larval mortality rates (Fig. 1b) and slightly but significantly reduced adult body mass (Fig. 3a). By contrast, *C*. *stigmatica* seems to be better adapted to late-season conditions, as its larval mortality is less dependent on day length, despite stronger developmental acceleration, and adults are not smaller under short-day conditions, perhaps owing to a more frugal metabolism. There is ample evidence that many temperate insects accelerate development and/or accumulate reserves for successful overwintering^22,25,60–62^.

### Repeatability of results

This is the first study to test the effects of temperature and photoperiod on the immature development in *C*. *stigmatica*, and we are not aware of any published material that could be compared with our findings. However, our experiments are carried out during two consecutive years and it is important to note that the main results are essentially replicated in the second year (Figs 2 and 3), in spite of different rearing conditions. Although *C*. *rubiginosa* was not previously studied in a similar experimental setting either, there exist estimates of its development time under laboratory and field conditions.

Egg and pupal durations measured by us in *C*. *rubiginosa* are close to those reported by Ward and Pienkowski^11^, but larval development times and adult body masses differ dramatically. The population studied by these authors originated from Virginia (USA), and larvae were reared on potted thistles (in the present study, *C*. *rubiginosa* was reared on fresh cut leaves of burdock) under a 13-h and 17-h photophase. Despite higher survival rates, larval development in their experiments was slower (e.g., 15.2 days at 26.6 °C and 35 days at 17.8 °C – cf. Table 2) and adults were smaller (treatment group means ranged from 10.5 to 14.4 mg – cf. Fig. 3a). This discrepancy is intriguingly reminiscent of our findings with *C*. *stigmatica* reared on cut leaves vs. living plants, but it is premature to draw parallels because of many possible confounding factors involved. The same authors showed^11^ that *C*. *rubiginosa* developed in the field significantly faster than could be expected from their experimental estimates.

Also in Virginia, Spring and Kok^8^ found that the period from hatching to adult emergence under field conditions was about 22 days on thistles and 23.8 days on burdock. Mean temperature during that period was approximately 22 °C. Thus, development times recorded by Ward and Pienkowski^11^ are anomalously long, whereas our laboratory estimates of postembryonic development time in a Russian population of *C*. *rubiginosa* fed with cut burdock leaves (Table 2) are in good agreement with the phenology of this species in Virginia. Body mass of freshly eclosed, unfed adults in our experiments (Fig. 3a) is also consistent with body mass of field-collected and overwintered adults from Virginia (17.6 and 24.4 mg for males and females, respectively^11^). In addition, field development times and body masses suggest that defensive reactions from host plants, if any, do not significantly inhibit larval development and growth in *C*. *rubiginosa*. A possible alternative explanation for the discrepancy between the two rearing methods may be that potted and wild-growing plants differ in nutrient content. So far, the causes of prolonged development and small body size in both tortoise beetle species when reared on living plants in the laboratory remain unknown.

## Methods

### Collection and rearing

Experiments were carried out in 2017 and 2018 with the first-generation progeny of beetles collected in the field. Overwintered adults of *C*. *rubiginosa* and *C*. *stigmatica* were hand-collected by inspecting their potential host plants in Bryansk, Russia (53°15′N, 34°17′E) on May 19–24, 2017. The latter species was also collected on May 10–12, 2018, for an additional experiment. Although various species of Asteraceae were examined, *C*. *rubiginosa* was exclusively found on *Arctium* spp. and *C*. *stigmatica*, on *T*. *vulgare*. The total number of parental adults transported to the laboratory in St Petersburg was 15 individuals of *C*. *rubiginosa*, 82 individuals of *C*. *stigmatica* in 2017, and 41 individuals of *C*. *stigmatica* in 2018. Their sex was not determined because this procedure is extremely difficult without dissection. In the laboratory, beetles were kept in groups of 6–10 in 250 ml transparent plastic containers with ventilation holes in the lid and moist sawdust on the bottom. Containers were placed in an environmental chamber maintained at 23–24 °C under a long-day photoperiod of 18 L:6D Beetles were daily supplied with fresh cut leaves of the same plant species they had been collected from.

Eggs were carefully excised with the underlying leaf fragment or, in the case of *C*. *rubiginosa*, which did not always oviposit on the leaves, detached from container walls using a sharp razorblade. Collected eggs were transferred with a moistened paintbrush to small plastic Petri dishes (40 mm in diameter), which in turn were placed into larger dishes (100 mm in diameter) on a layer of damp cotton wool to avoid desiccation of the eggs. Egg development was monitored daily, and, when leaf fragments deteriorated, fresh ones were added to prevent future hatchlings from starvation. On the day of hatching, each larva was carefully picked with a blunt preparation needle and transferred to a freshly cut leaf fragment that was placed into a 4 cm^3^ plastic cup with ventilation holes in the plug and a moistened piece of paper towel on the bottom. Each plastic cup usually housed 3 or 4 larvae (seldom 2 or 5, which depended on the number of hatchlings available). Larvae and pupae were also checked daily. Food and paper towel were changed as needed. Pupae were detached using forceps and transferred to 250 ml plastic containers where they were laid on a moistened cotton wool layer.

Relative humidity in larval cups during the 2017 experiment was maintained close to 100%, which appeared to be suboptimal to *C*. *stigmatica* (see below). However, it was not possible to maintain a lower humidity because cut leaves quickly withered and dried out. Therefore, an attempt was made at rearing *C*. *stigmatica* larvae on living plants. In October of 2017, tansy seeds were collected in the natural habitat of this beetle in Bryansk. The seeds were dried at room temperature and stored at 4 °C. In March of 2018, the seeds were sown in commercially available potting soil in indoor boxes under Dulux L 55 W/830 fluorescent lamps (Osram GmbH, Germany). Early in May, seedlings were individually transplanted to 500 ml plastic glasses half-filled with potting soil and covered with a paper sheet that was fastened to the rim by means of a rubber band. Newly hatched larvae of *C*. *stigmatica* were transferred to these tansy seedlings, 6–10 larvae per plant, and kept there until pupation. In all other respects, the rearing procedure was the same as in 2017.

### Experimental design

Eggs laid during the previous 24-h period were randomized among ten experimental regimens: five constant temperatures (16, 19, 22, 25, and 28 °C) and two photoperiods (short-day 12 L:12D and long-day 18 L:6D). Throughout the entire immature development, all individuals remained in the regimens to which they had initially been assigned. Eggs laid by both species before the arrival to the laboratory in 2017 and all eggs of *C*. *stigmatica* in 2018 were incubated until hatching in the chamber where parental adults were kept (23–24 °C, 18 L:6D), and newly hatched larvae were allocated among the same ten experimental regimens. Eggs, larvae, and pupae were monitored daily, and the date and time of hatching, pupation and adult emergence were recorded. Adults were weighed on a Discovery DV215CD electronic balance with 0.01 mg precision (Ohaus Corporation, USA) on the day of eclosion. Sex was determined by dissection.

Temperature in the environmental chambers was maintained to ±0.1–0.5 °C via a software-controlled balance of heating and cooling (RLDataView 1.03; Research Laboratory of Design Automation, Taganrog, Russia) and automatically recorded every 10 s. Actual rearing temperatures slightly deviated from the set values and are given in Tables 2 and 3, but for convenience we refer to them as integers throughout the text.

### Statistical analyses

Statistical analyses were carried out in R version 3.5.1 with RStudio^63,64^. In all analyses, temperature was treated as a continuous independent variable and all other factors (photoperiod, sex and experimental year) were treated as categorical predictors. No random-effects structure could be identified because rearing groups consisted of randomly picked individuals. A preliminary inspection of data showed no relationship between rearing-group size and either development time or adult body mass, and so it was unlikely that group rearing or variation in survival rates could have introduced significant confounding to our results.

Survival of immature stages and adult sex ratio under experimental combinations of temperature and photoperiod were analyzed by fitting generalized linear models (GLMs) with a logit link and binomial error structure. In addition, for illustration purposes, survival rate in each regimen was expressed as a percentage of individuals successfully completing a given stage ± binomial s.e.

Egg, larval and pupal developmental rates were calculated for each individual as inverse durations of the corresponding stages (days^−1^). The effects of temperature, photoperiod, sex, experimental year and species identity on developmental rate and body mass were analyzed using the generalized least-squares (GLS) method under restricted maximum likelihood with different variances for each combination of factors^65^. Analyses were performed using the gls() function in the nlme package^66^. Significance of differences was determined with *F*-tests based on type I (sequential) sum of squares. Model assumptions of homoscedasticity, linearity, and normality of residuals were verified by inspection of raw and standardized residuals plots.

The responses of developmental rate (*R*) to temperature (*T*) were described in greater detail by means of linear regression equations of the form^46^ *R* = *a* + *bT*. GLS models were re-run with temperature as the single explanatory variable for all subsets of data where the response significantly (*p* < 0.0001) differed between the levels of a categorical predictor. The intercept (*a*) and slope (*b*) were thus obtained separately for each combination of species, developmental stage, photoperiod and experimental year. These *a* and *b* values were then used to calculate two biologically meaningful parameters: the lower temperature threshold for development LTT = −*a*/*b* and the sum of degree-days SDD = 1/*b*.

## Supplementary information

Supplementary Figure 1

Supplementary Tables 1 and 2

Dataset 1

## Data Availability

The datasets generated during the current study are available as online Supplementary information.
