# Supplementary material for: Effects of Temperature and Photoperiod on the Immature Development in Cassida rubiginosa Müll. and C. stigmatica Sffr. (Coleoptera: Chrysomelidae)
Source: Sci Rep. 2019 Jul 11;9:10047. doi: 10.1038/s41598-019-46421-3 (PMC6624315; doi:10.1038/s41598-019-46421-3)
Supplement: Supplementary file 1 — Supplementary Figure 1 [file 41598_2019_46421_MOESM1_ESM.pdf]

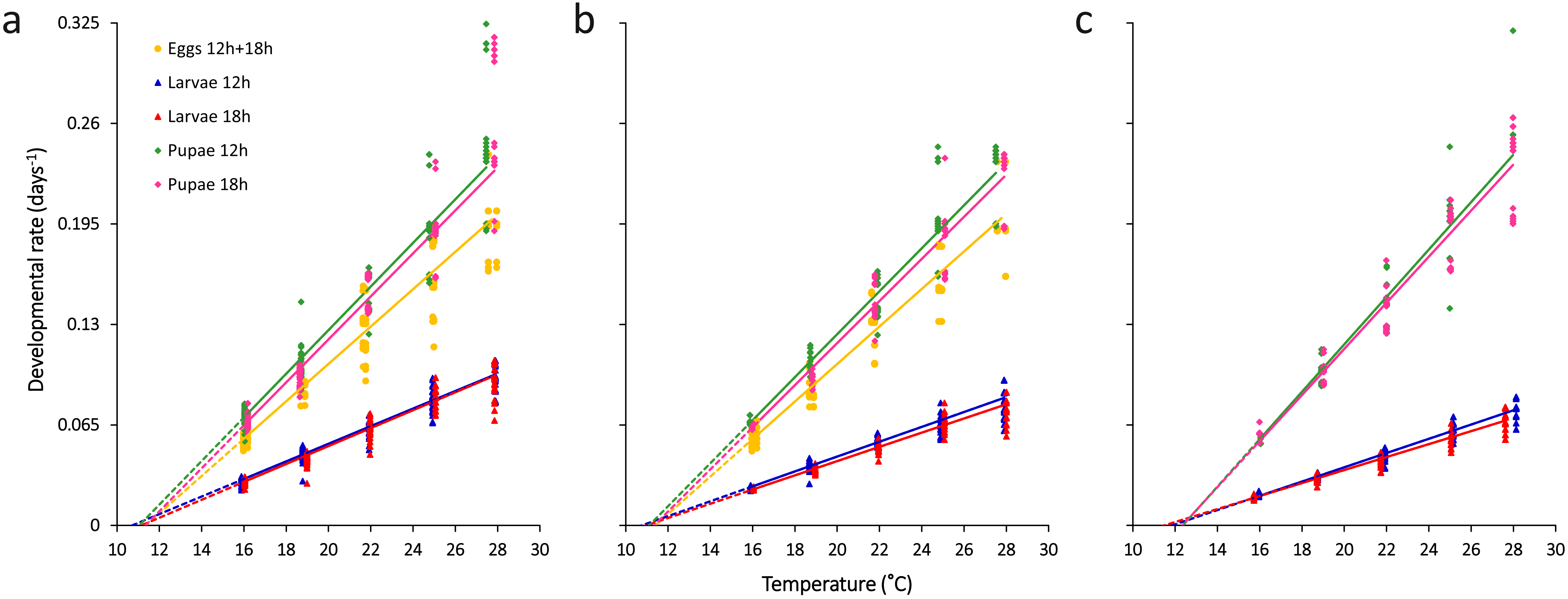

Supplementary Fig. S1. Thermal reaction norms for immature development in (a) *Cassida rubiginosa* and (b, c) *C. stigmatica* under two photoperiods, the latter species studied during two consecutive years (b: 2017, c: 2018). Regression lines are plotted based on generalized least squares (GLS) model parameters fit by restricted maximum likelihood (REML). Regression lines are the same as in Fig. 2. Data points represent individual developmental rate values (there is usually more than one observation per point).
