# Supplementary material for: Effects of Temperature and Photoperiod on the Immature Development in Cassida rubiginosa Müll. and C. stigmatica Sffr. (Coleoptera: Chrysomelidae)
Source: Sci Rep. 2019 Jul 11;9:10047. doi: 10.1038/s41598-019-46421-3 (PMC6624315; doi:10.1038/s41598-019-46421-3)
Supplement: Supplementary file 2 — Supplementary Tables 1 and 2 [file 41598_2019_46421_MOESM2_ESM.docx]

**Online Supplementary Information**

Supplementary Table S1. Larval and pupal development time (mean ± SD, days) in male and female *C. rubiginosa* under five constant temperatures and two photoperiods

| Day length (h) | Temperature (°С) | | Sex | Larvae | Pupae | *N*^†^ | f/m sex ratio |
| --- | --- | --- | --- | --- | --- | --- | --- |
|  | Set | Real |  |  |  |  |  |
| 12 | 16 | 15.9 | f | 34.6±2.47 | 13.8±0.73 | 26 | 1.4 |
|  |  |  | m | 34.2±2.77 | 14.5±1.36 | 19 |  |
|  | 19 | 18.7 | f | 22.0±2.87 | 9.8±0.81 | 24 | 0.9 |
|  |  |  | m | 20.9±1.22 | 9.8±0.67 | 28 |  |
|  | 22 | 21.9 | f | 15.8±0.82 | 6.6±0.51 | 15 | 0.6 |
|  |  |  | m | 15.6±1.54 | 6.5±0.59 | 27 |  |
|  | 25 | 24.9 | f | 12.2±0.88 | 5.2±0.41 | 38 | 1.9 |
|  |  |  | m | 12.0±1.00 | 5.1±0.49 | 20 |  |
|  | 28 | 27.7 | f | 11.2±1.00 | 4.0±0.35 | 21 | 0.8 |
|  |  |  | m | 10.6±0.83 | 4.1±0.50 | 26 |  |
| 18 | 16 | 16.1 | f | 35.4±2.41 | 14.5±0.93 | 25 | 0.8 |
|  |  |  | m | 34.4±1.74 | 14.6±0.74 | 31 |  |
|  | 19 | 18.9 | f | 23.7±2.48 | 10.4±0.55 | 37 | 1.2 |
|  |  |  | m | 23.1±1.26 | 10.4±0.54 | 30 |  |
|  | 22 | 21.9 | f | 15.7±1.14 | 6.6±0.49 | 31 | 0.9 |
|  |  |  | m | 15.7±1.45 | 6.7±0.49 | 36 |  |
|  | 25 | 25.0 | f | 12.3±0.63 | 5.2±0.32 | 38 | 1.2 |
|  |  |  | m | 11.9±0.80 | 5.3±0.39 | 31 |  |
|  | 28 | 27.9 | f | 10.7±1.23 | 4.0±0.48 | 32 | 1.2 |
|  |  |  | m | 10.4±0.81 | 4.0±0.45 | 26 |  |

^†^Sample sizes are equal because only individuals that survived to the adult stage were taken into account.

Supplementary Table S2. Larval and pupal development time (mean ± SD, days) in male and female *C. stigmatica* under five constant temperatures and two photoperiods

| Year | Day length (h) | Temperature (°С) | | Sex | Larvae | Pupae | N^†^ | f/m sex ratio |
| --- | --- | --- | --- | --- | --- | --- | --- | --- |
|  |  | Set | Real |  |  |  |  |  |
| 2017 | 12 | 16 | 15.9 | f | 40.9±2.43 | 15.1±0.13 | 6 | 0.7 |
|  |  |  |  | m | 40.9±1.31 | 14.7±0.54 | 9 |  |
|  |  | 19 | 18.7 | f | 24.6±1.82 | 9.6±0.48 | 10 | 0.6 |
|  |  |  |  | m | 25.8±3.16 | 9.5±0.55 | 16 |  |
|  |  | 22 | 21.9 | f | 18.0±0.88 | 6.8±0.48 | 26 | 1.2 |
|  |  |  |  | m | 17.9±0.80 | 6.9±0.53 | 21 |  |
|  |  | 25 | 24.9 | f | 15.2±1.28 | 5.0±0.41 | 22 | 0.7 |
|  |  |  |  | m | 15.1±1.00 | 5.2±0.30 | 32 |  |
|  |  | 28 | 27.7 | f | 12.9±1.22 | 4.2±0.28 | 23 | 0.6 |
|  |  |  |  | m | 12.1±1.22 | 4.6±0.50 | 37 |  |
|  | 18 | 16 | 16.1 | f | 42.4±1.25 | 15.4±0.51 | 8 | 4.0 |
|  |  |  |  | m | 42.7±1.41 | 15.9±0.21 | 2 |  |
|  |  | 19 | 18.9 | f | 28.2±1.06 | 10.7±0.46 | 13 | 1.6 |
|  |  |  |  | m | 27.4±1.79 | 10.5±0.50 | 8 |  |
|  |  | 22 | 21.9 | f | 19.9±0.80 | 7.0±0.49 | 37 | 1.2 |
|  |  |  |  | m | 19.7±1.31 | 7.0±0.40 | 30 |  |
|  |  | 25 | 25.1 | f | 15.3±0.75 | 5.3±0.39 | 27 | 1.0 |
|  |  |  |  | m | 15.0±1.11 | 5.3±0.44 | 28 |  |
|  |  | 28 | 28.0 | f | 12.8±1.21 | 4.5±0.48 | 30 | 0.9 |
|  |  |  |  | m | 12.8±1.14 | 4.5±0.46 | 32 |  |
| 2018 | 12 | 16 | 16.0 | f | 50.1±3.84 | 17.3±0.35 | 4 | 1.0 |
|  |  |  |  | m | 52.1±1.62 | 18.1±0.93 | 4 |  |
|  |  | 19 | 18.8 | f | 31.8±1.66 | 9.9±0.46 | 11 | 0.8 |
|  |  |  |  | m | 31.9±1.75 | 10.1±0.58 | 13 |  |
|  |  | 22 | 21.9 | f | 22.2±1.47 | 7.0±0.51 | 29 | 2.2 |
|  |  |  |  | m | 22.1±2.04 | 7.1±0.61 | 13 |  |
|  |  | 25 | 25.1 | f | 16.2±1.15 | 5.1±0.37 | 19 | 1.3 |
|  |  |  |  | m | 15.6±0.88 | 5.2±0.69 | 15 |  |
|  |  | 28 | 28.1 | f | 13.4±0.79 | 4.1±0.47 | 22 | 1.0 |
|  |  |  |  | m | 13.0±0.76 | 4.1±0.22 | 22 |  |
|  | 18 | 16 | 15.8 | f | 54.3±3.44 | 17.8±1.16 | 4 | 0.7 |
|  |  |  |  | m | 55.3±4.39 | 16.8±1.15 | 6 |  |
|  |  | 19 | 18.8 | f | 33.1±1.74 | 10.0±0.59 | 20 | 1.1 |
|  |  |  |  | m | 32.7±2.59 | 10.2±0.57 | 19 |  |
|  |  | 22 | 21.8 | f | 24.2±2.08 | 7.1±0.49 | 24 | 1.3 |
|  |  |  |  | m | 23.6±1.29 | 7.4±0.62 | 18 |  |
|  |  | 25 | 25.0 | f | 18.3±1.59 | 5.2±0.45 | 15 | 0.7 |
|  |  |  |  | m | 17.4±0.81 | 5.1±0.39 | 21 |  |
|  |  | 28 | 27.7 | f | 15.0±1.55 | 4.1±0.40 | 22 | 0.9 |
|  |  |  |  | m | 14.1±0.88 | 4.3±0.48 | 24 |  |

^†^Sample sizes are equal because only individuals that survived to the adult stage were taken into account.
